# What can implementation science offer civil society in their efforts to drive rights-based health reform?

**DOI:** 10.1186/s41256-023-00284-4

**Published:** 2023-01-17

**Authors:** Diya Uberoi, Tolulope Ojo, Abi Sriharan, Lincoln Lau

**Affiliations:** 1 Faculty of Law, McGill University, 3644 Peel St, Montreal, QC H3A 1W9 Canada; 2 Dalla Lana School of Public Health, University of Toronto, 155 College Street, Toronto, M5T 3M7 Canada

**Keywords:** Right to health, Access to medicines, Implementation science, Civil society

## Abstract

Over the years, civil society organizations (CSOs) have made tremendous efforts to ensure that state policies, programmes, and actions facilitate equitable access to healthcare. While CSOs are key actors in the realization of the right to health, a systematic understanding of how CSOs achieve policy change is lacking. Implementation science, a discipline focused on the methods and strategies facilitating the uptake of evidence-based practice and research can bring relevant, untapped methodologies to understand how CSOs drive health reforms. This article argues for the use of evidence-based strategies to enhance civil society action. We hold that implementation science can offer an actionable frame to aid CSOs in deciphering the mechanisms and conditions in which to pursue rights-based actions most effectively. More empirical studies are needed to generate evidence and CSOs have already indicated the need for more data-driven solutions to empower activists to hold policymakers to account. Although implementation science may not resolve all the challenges CSOs face, its frameworks and approaches can provide an innovative way for organizations to chart out a course for reform.

## Introduction

When goals such as societal health move from aspiration to the status of a fundamental right, great strides can be made in social equity. Civil society in this process, refers to the wide variety of groups that function outside government to advocate for the rights of disadvantaged populations. They include formal non-governmental organizations (NGOs), faith-based groups, and activists; each of whom are central to driving social change and rights-based reform [1]. Over the years, these actors have been instrumental, employing several rights-based strategies such as advocacy, litigation, and citizen empowerment to push for more equitable access to health services and treatment [2]. In the early 2000s, when the HIV crisis was at its peak in sub-Saharan Africa, CSOs led the advancement of affordable antiretroviral (ARV) drugs. Their efforts not only achieved a dramatic global reduction in the price of HIV drugs, but also saw corporations, governments, and international organizations shift toward advocating universal access to ARV treatment [3]. Similarly in Kenya, the combination of litigation and advocacy fueled through CSO networks led to the revision of the 2008 Counterfeit Act, which had prohibited HIV persons from accessing affordable generic drugs [4].

Because rights-based change can uproot systemic social issues, CSOs often face political, social, cultural, and financial challenges. In several low- and middle-income countries (LMICs), governments have imposed restrictions that limit the ability of CSOs to access national and foreign funding [5]. Shifts in power among political parties also impact CSOs ability to drive rights-based reform in LMICs [6]. CSOs additionally face internal structural challenges such as unskilled personnel, minimal and often sequestered knowledge, and lack of competent supervision, each of which can undermine their efforts in rural, hard to reach areas [7]. There are several studies that highlight such issues in CSO structures and operations, but few studies have offered a view for improvement, especially with regards to identifying and improving these weak spots in CSO functionality. Research in this area is crucial, as human rights scholarship, has already called into question the efficacy of using rights-based strategies in the first place. Studies from Brazil and Colombia, for example, show how rights-based litigation undermines equity when claims are brought with personal intention rather than broader health goals in mind [8–10]. A constructive approach to such outliers is to gain insight from these cases in order to understand and strengthen the full use of rights-based strategies while further strengthening CSO capability.

This perspective article argues for the use of evidence-based strategies to enhance civil society action. Implementation science, a discipline focused on the systematic study of methods and strategies to facilitate the uptake of evidence-based practice, provides a well-founded means to evaluate the challenges CSOs face when pursuing rights-based strategies [11]. With this in mind, this article proffers that the tools utilized in implementation science can guide CSOs in understanding their real-world decisions. Although a full review of all implementation science methods is outside the scope of this article, we highlight potentially useful aspects that can be leveraged to better understand and guide CSO action towards improved health outcomes. First, we consider the role of CSOs in advancing equitable access to health care and some of the limitations they face. We then turn to the definition of implementation science and how it can facilitate the uptake of research evidence into practice. Then, applying aspects of implementation science to the study of civil society activity, we consider its potential in explaining and informing CSO efforts to realize the right to health for all.

## CSOs as drivers of health reform

The reduction of health disparities is central to the pursuit of equity—whereby all individuals and communities have an equal opportunity to be healthy [12]. The right to health requires that governments not only prioritize needs of the disadvantaged in their policies, but use their available resources to ensure equitable access to health services and treatment for all [13]. Over the years, CSOs advocacy programmes and litigation strategies have been instrumental in reforming policies to meet the needs of disadvantaged populations. In areas not solely limited to access to medicines, reform of blood banks, drug policies, tobacco control, and reproductive health, CSO effort has been crucial in shifting policies toward a higher global standard [14]. As mentioned in the introduction, it was the combination of litigation and lobbying efforts by CSOs that catalyzed the availability of more affordable ARV treatment in South Africa. The social and political engagement garnered by the Treatment Action Campaign (TAC) in 2002, led to the country’s Constitutional Court holding the government accountable for its denialism and inaction in dealing with HIV [15].

Today, CSOs around the world have been able to galvanize around the right to health in similar ways. In India, CSOs have been relentless in their quest, combining a variety of tactics to ensure the availability of affordable care. This is particularly evident in cases of access to medicines, where CSOs have used litigation and advocacy to advance generic drug manufacturing resulting in lower drug prices, increased availability and ultimately, more equitable access to health services and treatment [16]. Today, India is recognized as the “pharmacy” of the developing world, in part because of the work of CSOs leveraging the right to health to advocate for access to medicines as a human right [17].

While India and South Africa illustrate examples where CSOs were able to successfully leverage rights-based strategies, CSOs attempts to improve access to treatment, have met with a unique set of challenges. Several studies suggest that CSO efforts may be hijacked by personal interests such as wealthy individuals seeking access to specific drugs—thereby undermining broader public gains. Studies from Brazil and Colombia, for example, show that most individual claims are brought by wealthy individuals seeking access to expensive medical treatments and high-cost drugs not listed on government formularies. The skewing of judicially determined cases and settlements toward such individuals distorts the ability of CSOs to actualize equitable access for the most marginalized [8, 18].

The gaps in civil society efforts to actualize rights-based actions provide an opportunity to consider opportunities for improvement. Currently there is a dearth of research that systematically studies the resources, structures, and strategies civil society employ in their efforts to realize equitable health for all [19]. While the development community has been actively engaged in efforts to strengthen civil society efforts, empirical evidence to measure the impact of their work has been limited [20, 21]. Studies show how, lack of methods and tools for monitoring outreach, can keep CSOs uninformed around the uptake of their work. Researchers have pointed out that a key challenge in improving civil society function is the development adequate methodologies and tools to objectively understand and guide civil society action [22, 23]. Existing studies are mainly case studies and predominately seek to describe the state of civil society, its external environment and contributions to policy reform and development [21, 22]. With tools to guide their everyday activities, CSOs may be able to better decipher the conditions in which their rights-based actions may succeed. Increasingly, civil society actors are recognizing the need for data driven solutions to empower activists to hold policy makers and service delivery organizations to account. For example, in South Africa a coalition of CSOs including TAC established “Ritshidze”, a community led project that uses systematic data collection to monitor failures in quality HIV and TB service delivery to empower communities to demand for improved access to HIV services in line with evidence-based solutions [24].

With its focus on uncovering context and decoding replicable commonalities, implementation science offers a useful, practicable frame of analysis and action. The following sections consider the growth of implementation science as a field and its application to the systematic study of CSOs.

## Defining implementation science and understanding its application to Civil Society efforts in driving health reform

Although several definitions exist, implementation science can be broadly understood as the “application and integration of research evidence into practice” [25]. It aims to identify and surmount the barriers impeding the translation of knowledge into practice [26]. In this respect, implementation science moves beyond understanding ‘what works’ to address the contexts and mechanisms that *impact* what works, where, and why [27]. In other words, the field looks beyond traditional clinical research, to understand and conceptualize context as a determinant of implementation outcomes [28]. While this includes a focus on the providers of health services themselves, it equally recognizes the role that interactions between institutions, political interests and policy play in driving health outcomes [29–31]. As such, implementation research requires multidisciplinary research teams which can include anthropologists, sociologists, health services researchers, behavioral scientists, organizational scientists, administrators and others [29]. With its ability to highlight the gap between evidence and action, implementation science allows for a comprehensive understanding of how policies can be better designed and implemented. Implementation science offers researchers a range of theories, models and frameworks (TMFs), to identify and describe potential barriers and facilitators associated with the uptake of evidence across a variety of spectrums. While these TMFs have been described in previous reviews [32], they primarily include: (1) Process models that describe the steps or processes for translating research into practice which can illuminate the steps necessary for implementation [33, 34]; (2) Determinant frameworks, which describe factors influencing outcomes, that can be useful in understanding the contexts in which interventions and strategies may succeed or fail [33]; and (3) Evaluation frameworks which can help assess the impact that health interventions may have on the ground [35, 36]. By leveraging its TMFs, researchers and practitioners can position implementation research to better guide the development of more evidence-informed policies.

The following discussion, highlights the four ways implementation science TMFs can contribute to civil society research and practice:

### Application of Implementation Science Frameworks to understand determinants of success

If CSOs are to maximize their efforts in achieving more equitable health policies in LMICs, it is vital that their rights-based strategies be grounded in evidence. This is particularly true in the global health policy space, where globalization has resulted in a complex new landscape with shared human rights frameworks that CSOs must understand and negotiate at the national and international level [37, 38]. In this respect, a determinant framework, which highlights contextual factors as indicative of programmatic success, may be particularly useful in highlighting the way political and legal realities can impact CSOs strategies. As Heywood explains, there are contextual prerequisites that either facilitate or frustrate CSOs use of rights-based strategies toward improved population health outcomes [39]. For example, a critical factor for CSO success in India and South Africa is the legal environment, where socio-economic rights—including the right to health—are directly enforceable through courts. Laws around who has the right to bring an action to court are relatively relaxed in these countries, allowing for an environment that encourages policy reform via litigious CSOs [40]. Some evidence suggests that in authoritarian regimes, confrontational rights-based approaches are not effective in bringing about structural transformation, as they provoke intense backlash of the kind that makes it impossible for CSOs to operate [6]. With a determinant framework in hand, CSOs may be able to better navigate when and how to tailor their strategies and approaches in a way that can ensure a stable working relationship with, and compliance from, the state.

Another factor that greatly influences CSO impact is access to funding. At the national level, governments in LMICs often limit the national and foreign funding available for CSO operations in the field of human rights and democratic actions. In navigating such terrain, CSOs must take into account continually shifting factors that can enhance or undermine their strategies. Evidence suggests that donors are more likely to support organizations on the rise over more established groups [41]. TAC, for example, secured big bilateral contracts from foreign donors at the height of its HIV work but saw a sudden shift when improvements around the AIDs epidemic became evident. While TAC anticipated such shifts in funding, it did not expect to have difficulty in finding new donors. A better understanding of the mechanisms influencing external funding, could allow CSOs to potentially reorganize and prioritize their strategies to avert future financial challenges. For example, where external funds may not be available to finance litigation and advocacy efforts, CSOs may want to prioritize their community awareness efforts which may be more readily funded. Examining CSO impact without adequate consideration for these legal and financial factors is likely to result in flawed analysis and conclusions. Determinant frameworks can help identify external policies, incentives, and available resources- to highlight global and national actors, institutions, and interests influencing CSOs ability to drive reform [28, 33, 42].

Implementation science frameworks that explore organizational determinants of success may provide such organizations with relevant insights on how their internal structures and processes either support or hinder their intended outcomes [43]. Not all organizations have the capacity or ability to coordinate themselves successfully. A growing number of studies suggest that CSO networks are being hampered by organizational issues, as they don’t necessarily know how to plan for growth [44]. In Mexico, for example, CSOs have struggled to sustain growth as lack of organization has undermined their performance [44]. Therefore, implementation science-driven organizational theories that examine culture, climate and leadership can further provide relevant learnings for explaining how CSO structures impact their operations and executions of strategy. For example, an organizational readiness assessment can identify and categorize factors such as access to funding, political leadership and networks with other CSOs—which may contribute to CSOs’ capacity to drive change. Similarly, in translating advocacy programs from one setting to another, a process model can be beneficial to direct the steps for planning and adapting such efforts in new settings [34].

It is important to note that because existing implementation science frameworks and models were developed within the contexts of high-income countries; they may require a fundamentally different approach to fit the needs of LMICs. Even then, implementation science’s potential in low resource settings should not be overlooked: equity requires novel solutions that ensure that research results are translated into routine practice and benefit the largest possible number of people [45].

### Prioritization of strategies to improve outcomes

When studying CSOs actions and outcomes, CSOs can use implementation science’s rich set of theories, models and, frameworks, to understand the conditions in which certain rights-based strategies could be prioritized over others [46–49]. A central goal of implementation science is the selection of strategies to improve outcomes. In implementation science, context is regarded as the most important factor in determining if, how, or why a practice or intervention is successfully implemented. Depending on the context, a set of strategies may lead to better outcomes compared to others. Therefore, implementation science may help CSOs understand how to best target desired outcomes with effective strategies. Looking to access to medicines efforts, where litigation may have been the first resort of CSOs in the context of HIV medicines, current realities of a global epidemic and increased pushback from governments around rights-based work could suggest prioritizing other complementary actions [50].

### Strengthening CSO ability to engage with relevant stakeholders

A crucial aspect of implementation science is identifying appropriate strategies in collaboration with relevant stakeholders [33]. With relevant tools in hand, CSOs may be able to leverage their collaborative strategies more successfully. Civil society is steadily gaining relevance as co-creators of policy priorities with government. Their inclusion in the policy making process, however, has historically been fraught with problems including tokenism, corruption and intimidation of local actors [51]. Driving health reform requires collaborative engagement across stakeholders (civil society, communities, and policymakers) and implementation science TMFs can provide tools to guide engagement in a way that builds consensus among CSOs and their networks.

Stakeholder networks at the international and national level are a crucial, but an overlooked factor in driving reform. When communities mobilize, the power of rights-based approaches to induce change becomes increasingly apparent. For example, Peru-Mujer, a CSO that employs legal literacy as a tool for women’s empowerment in Peru, leveraged its vast network of community-based promoters of women’s reproductive rights, to support their clients in demanding protection of their rights from the state. The significant outreach and collaboration of the community-based promoters accorded them an advantage in the eyes of the Ministry of Finance where other, larger organizations had failed [52].

### Supporting CSOs to evaluate their own performance

Ultimately, however, rights-based reform still depends on CSOs ability to appraise the effectiveness of their strategies. Rigorous evaluation is necessary to ensure that evidence in advocacy practice will be translated into change [53]. Evaluation frameworks can be used to provide a structure for CSOs to appraise their own effectiveness [35]. The work of TAC stands as a case in point. Often cited as a model of a successful social movement, application of its strategies by other groups seeking to champion demands for greater social equity has been limited [54]. While some organizations have yet to explore networks or rely heavily on the conscience of human rights advocates, TAC’s success is partly attributable to the way it reorganized itself along a different approach, structuring advocacy activities via people living with HIV. Focusing on educating HIV-vulnerable people and the poor about the science of HIV, TAC became the first HIV/AIDS organization to practice ‘treatment literacy’ in a low-resource setting [55]. TAC’s focus on building an advocacy culture based on necessity rather than conscience created a strong, decentralized force that complemented their legal strategy. The growth of TAC and its struggles holds lessons for burgeoning civil society organizations. The global political demand to support HIV/AIDS activism in the 1990s was accompanied by increased global financial resources, leading to an unprecedented growth in size and costs for TAC. Ultimately, this led to a growing dependence on donors leaving TAC vulnerable to development aid policies and standards including complex management systems, which impacted the grassroots organization of communities that set up TAC for success in the first place [56]. Through assessment of successes, failures and insights gained from established NGOs like TAC, newer organizations will be able to better prioritize their needs.

The challenges CSOs face when pursuing policy change present several complexities, but with examination through an implementation science lens, researchers and practitioners will be better equipped to tailor CSOs strategies toward greater equity [19].

## Conclusions

CSOs play a key role in ensuring accountability for the right to health. However, numerous factors, from internal organization to the external legal and political environment can undermine their effectiveness. The dearth in social science research around these factors warrants further investigation into the failure and success of CSO strategies in pursuing policy change. Although implementation science will not resolve all the challenges CSOs face, drawing upon its approaches can provide an innovative way for organizations, public health activists, and researchers to systematically understand local determinants of success, identify context-specific challenges, and propose effective solutions toward tangible health policy reform.

## Abbreviations

CSOs: Civil society organizations
ARVs: Antiretrovirals
TAC: Treatment action campaign
TMFs: Theories, models and frameworks
LMICs: Low-and-middle income countries
